# A Deep Learning-based approach for forecasting off-gas production and consumption in the blast furnace

**DOI:** 10.1007/s00521-021-05984-x

**Published:** 2021-04-16

**Authors:** Stefano Dettori, Ismael Matino, Valentina Colla, Ramon Speets

**Affiliations:** 1 Scuola Superiore Sant’Anna ­ TeCIP Institute – ICT-COISP, Pisa, Italy; 2 Tata Steel, CA IJmuiden, The Netherlands

**Keywords:** Deep echo state networks, Long short-term memories, Recurrent neural networks, Industrial application, Blast furnace gas management, Forecasting

## Abstract

This article presents the application of a recent neural network topology known as the deep echo state network to the prediction and modeling of strongly nonlinear systems typical of the process industry. The article analyzes the results by introducing a comparison with one of the most common and efficient topologies, the long short-term memories, in order to highlight the strengths and weaknesses of a reservoir computing approach compared to one currently considered as a standard of recurrent neural network. As benchmark application, two specific processes common in the integrated steelworks are selected, with the purpose of forecasting the future energy exchanges and transformations. The procedures of training, validation and test are based on data analysis, outlier detection and reconciliation and variable selection starting from real field industrial data. The analysis of results shows the effectiveness of deep echo state networks and their strong forecasting capabilities with respect to standard recurrent methodologies both in terms of training procedures and accuracy.

**Supplementary Information:**

The online version contains supplementary material available at 10.1007/s00521-021-05984-x.

## Introduction

In the last decade, process industry and, in general, all the energy-intensive sectors, are facing increasingly complex economic challenges, due to the variability of the raw materials market, the enormous variability of the demand for goods and services (let us just consider what is happening in the ongoing period characterized by the COVID-19 pandemic) and the daily fluctuation of the energy market, in terms of cost and availability of electricity and fuels. Beyond the technical and economic difficulties, another aspect that was once marginal is now fortunately becoming more and more important, the sustainability of production and consumption of energy and goods. The industrial world has always been quite aware of the undeniable environmental impact of some specific processes, but the sensitivity toward those issues has emerged in the last decades, with the consequent search for new solutions that allow improving or renewing the nature of production process itself.

In this context, processes and related supervision and control systems are operated in a discontinuous way, trying to guarantee a constant quality of goods and services while operating in high-efficiency level in terms of energy consumptions. This huge variability leads processes and systems to exert in ever increasing nonlinear operating point, with a resulted difficulty in supervising and controlling behaviors, which were rarely faced in the past by process operator. The complexity of the new challenges puts a strain on current control systems due to several different issues. First, some systems may only be partially automated due to the restrictions dictated by specific application regulations. Secondly, the control of industrial systems is based on information and communications technology (ICT) systems and computing platforms, such as programmable logic controllers (PLCs) and industrial computers, characterized by computing performance that cannot always guarantee the implementation of particularly complex algorithms. The slow evolution of industrial systems is dictated, on the one hand, by the fact that these platforms are designed to guarantee the absolute safety and operation of the plants, also from the point of view of cybersecurity.

In the recent paradigm of Industry 4.0, artificial intelligence (AI) can take on the role of a lubricant to release or renew some consolidated mechanisms of the process industry engineering [1], in order not only to revive research in the field of the intelligent automation and supervision, but also to open a more constructive discussion for the study and implementation of approaches aimed at improving the socioeconomic and environmental sustainability of production processes.

In the last 5–10 years, in the industrial field, AI and especially machine learning (ML) have received an ever-increasing consensus and trust of the operators [2], given the plethora of works in both the academic and civil/industrial fields. This consensus is the result of numerous synergistic efforts by the academic and industrial worlds to make research and technology transfer more efficient. The results of these efforts show an increasingly accelerated advancement of the digitalization of civil and industrial contexts [3], thanks to the so-called *data-driven* methodologies, which are demonstrating, through their effectiveness, the importance of updating ICT systems also through more extensive sensorization of the plants and the relative collection and transmission of data. In the academic field, the direct relationship between the quantity, the quality in terms of variance of information and the effectiveness of the above techniques in terms of accuracy of predictions and system modeling is well known. This specific issue in the process industry is particularly delicate not only for economic reasons, but mostly due to technical difficulties. Just think about the challenge of measuring several state variables that are difficult to access due to hazardous environments, such as in the case of blast furnaces for the production of pig iron, in which temperatures exceed 1600 °C. Furthermore, in some processes, the materials and energy flow are not always easily measurable and, therefore, it may be difficult to bring into play particularly significant exogenous variables for increasing the prediction/modeling accuracy in time regression task.

In context of prediction and modeling of industrial processes is therefore important to select an adequate methodology to overcome the abovementioned issues. The most widespread methodology for modeling nonlinear time dynamics in the context of AI is undoubtedly the one based on the recurrent neural networks (RNN). RNNs parameters are identified through learning techniques that, in general, are based on backpropagation supervised methods. Several algorithms for training RNN architectures are proposed in the literature, such as backpropagation through time [4] and approaches based on the use of extended Kalman filter techniques [5]. The effectiveness of the algorithms depends mainly on the experience in the selection of the appropriate hyperparameters, the network architecture and the quality of the data, which are exploited for the training. Gradient descent-based algorithms are considered the standard solution for training RNNs, despite suffering from numerous problems such as exploding and vanishing gradients phenomena [6]. Some solutions to this problem have been proposed in the literature, such as methodologies based on numerical regularization techniques introduced by Pascanu et al. [7] and on appropriate heuristics for selection of the hyperparameters of the training algorithm [8]. In general, depending on the specific problem, the identification of a sufficiently stable and reliable model based on RNN techniques can be very complex. Despite RNNs are considered universal approximators, their application in time-critical applications is often impractical. The recent paradigm of reservoir computing (RC) offers valid solution for the problem and can be seen, among the different ML-based methodologies, as an enabler for an effective technology transfer. Reservoir computing has been introduced by Maass et al. [9] by introducing a RNN architecture called *liquid-state machine*.

In the context of industrial process modeling and forecasting, several RC methodologies have been presented in the literature, among which echo state network (ESN) are increasingly exploited and appreciated. An interesting related application has been presented by Wang [10] in which ESN and sparse AdaBoost forecast the electricity consumption in industrial areas. Bianchi et al. exploited ESN in combination with principal component analysis decomposition to forecast the short-term electric load in the power grid [11]. More recently, Zhang et al. [12] applied ESNs in combination with Jordan neural networks and least squares support vector machines in order to forecast short-term electric load and electricity price. In the context of process industry, Matino et al. presented a work related to the forecasting of blast furnace gas through ESN techniques [13], and Dettori et al. highlighted the effectiveness of AI methodologies for modeling energy transformation equipment in the industry [14]. Colla et al. extended the concepts by presenting the application of outlier detection and advanced variable selection to RC methodologies in industry [15]. Pan exploited ESNs in control application, within model predictive control structure with successfully results [16]. The ESN paradigm, extended to implement deep learning (DL) [17], has been successfully exploited in different domains. For instance, deep ESNs (DESN) have been recently applied by Kim and King [18] for time series prediction. For the same kind of application, Hu et al. [19] proposed an ensemble Bayesian DESN network model, whose flexible architecture allows overcoming some limitations of shallow ESNs related to their fixed architectures and difficulties in automatically determining the values of their hyperparameters.

This paper discusses the application of DL for forecasting of the energetic content and chemical characteristics of some process off-gases (POGs), which are produced and partially reused, in the integrated steelmaking route. Such forecasting models are components of a wider system aimed at optimizing the distribution of such gases among their consumers over a time horizon of 2 h by getting maximum value from their usage and avoiding wastes. In particular, this work proposes a comparison between deep echo state networks (DESN) and long short-term memories (LSTM) for modeling and forecasting the complex nonlinear behavior of the blast furnace (BF) process and of some related auxiliary units (i.e., hot blast stoves) as far as off-gas production and consumption are concerned. The novelties presented in this work are related to the application of DL methodologies and in particular DESN, for forecasting the energy contents of processes that are common in the steel industries, namely processes characterized by state variables that cannot be easily measured and poor available exogenous information.

The paper is organized as follows: Sect. 2 provides some theoretical background on DESNs and LSTMs; Sect. 3 presents the considered industrial application; Sect. 4 provides details of the developed models and exploited industrial datasets; Sect. 5 focuses on the obtained numerical results, while Sect. 6 provides some concluding remarks and hints for future work.

## The problem of forecasting Blast Furnace Gas production and consumption

The steelworks are energy-intensive industries, which are always committed to improve their energy and resource efficiency. In particular, within the integrated steelmaking route, which produces steel from virgin primary raw material (mainly iron ore and carbon), about 25% of the production costs are related to energy [24]. Achieving optimal exploitation of available energy sources is therefore of utmost importance and can lead to considerable cost savings, by thus contributing to keep sector competitiveness on the global market. POGs are a particular by-product of integrated steelworks. They are produced in some main production steps of the route: the coke ovens (a pre-processing stage for fossil Carbon), the blast furnace (BF), which is fed with carbon coke and sintered iron ore to produce pig iron, and the basic oxygen furnace, where pig iron is converted into steel through a decarburization and chemistry refinement process. POGs are rich in CO and H_2_ and thus have a significant net calorific value (NCV). Therefore, they are generally recovered and exploited as internal energy sources, fed to power plants to produce electricity or exploited to produce steam. However, POGs are not continuously generated: In some cases, their production is concentrated in limited time intervals and their features are not always constant, especially in terms of NCV. This fact poses not negligible problems for their optimal exploitation. Moreover, also POGs consumption can be discontinuous, as it is partly related to the production scheduling and it is subjected to a series of constraints linked to the complexity of the gas, steam and electricity distribution networks, which characterize each steelwork. In other words, in a given time interval many consumer processes can compete in POGs exploitation, although they are not always directly linked to all the POGs producers, which are active in the same interval. POGs can also be stored into gasholders, which, however, show limited capacities and dynamics and, thus, might not be capable of fully satisfy consumers demands and/or to store all the volume of gas produced by one single process. POGs over-production occurs when gasholders are full and is overcome by flaring the excess gas through the torches, but this represents a waste of a useful energy source and implies also CO_2_ emissions. On the other hand, POGs under-production leads to the need of exploiting natural gas to meet the unsatisfied energy demands, with a consequent costs increase.

The problem of optimal management and distribution of POGs in integrated steelmaking sites has already been faced in the literature, due to its importance in reducing costs and emissions. The problem to find the most suitable POGs distribution on a plant-wide basis is formulated as a single or multiple objective optimization problem and frequently solved via mixed integer linear programming (MILP)-based approaches, such as in the exemplar works of Kong et al. [25]. A further example is given by the work of Porzio et al. [26], who developed a decision support tool based on the application of flowsheeting models and multi-objective optimization approaches in order to find the optimal distribution of POGs. The same authors also extended the same tool also to the analysis of possible gas network modifications, which can further improve such distribution [27]. However, this tool exploits static models and does not consider the dynamics of POGs production and demand; therefore, the associated approach cannot be used for online POGs dynamic optimization. On the other hand, the possibility to forecast POGs production and consumption at least on a relatively short time horizon (e.g., a few hours) facilitate timely reaction of the system and support optimization over the whole time horizon.

In particular, as far BF gas (BFG) is concerned, some ML-based forecasting models can be found in the literature. For instance, Zhang et al. applied backpropagation neural networks [28], while Yang et al. applied improved least squares support vector machine and multiple linear regression [29, 30]. However, none of these models forecast the energetic value of BFG in terms of NCV, which is a fundamental information, as the distribution of this gas is not only based on the available volume flow, but also on the conveyed energy compared to the needs and requirements of the different potential gas consuming processes and utilities.

**Fig. 1:**
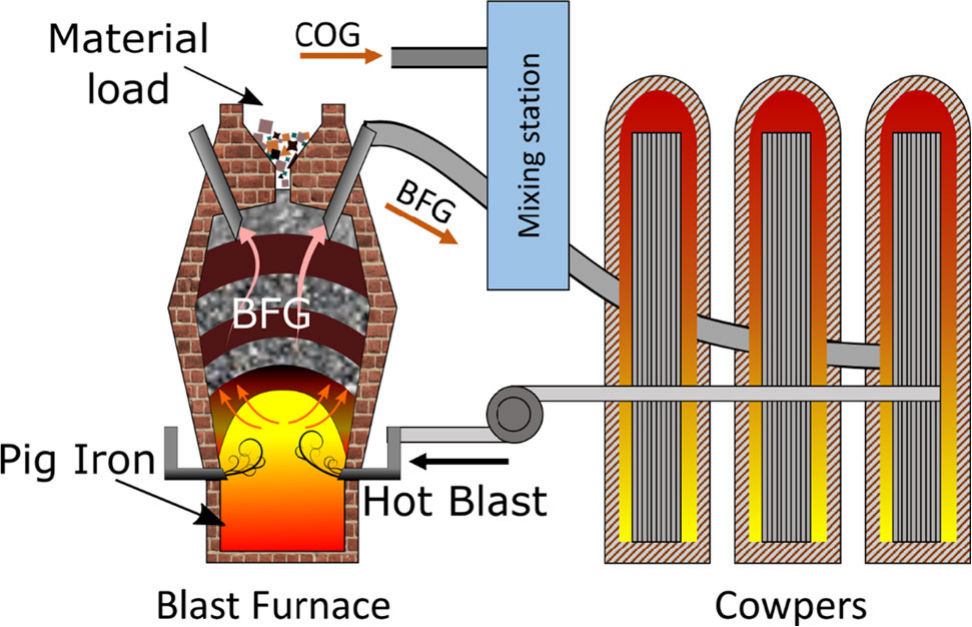
Blast furnace and Cowpers processes

The present work faces the problem of forecasting the amount and characteristics of the produced BFG on a future time horizon of 2 h by using a restricted number of process measurements and future knowledge of the process scheduling. Together with the prediction of the BFG production, the forecasting of BFG and coke oven gas (COG) consumption in the hot blast stoves (also named Cowpers) is also provided. In the BF process (which is schematically depicted in Fig. 1), air is firstly preheated and then blown inside the BF itself. Such preheating exploits combustion of BFG and other byproduct gasses typically available in some integrated steelworks, such as COG. Knowing in advance the BFG and COG consumptions of the Cowpers is a fundamental information in order to know which portions of these two POGs will be available for other consumers.

To sum up, the objective of the models is to predict the production of POGs, in particular their energy content in terms of NCV and volume flow, as well as their consumption in the Cowpers.

For the model design, real data provided by industrial partners were available and. in particular, a dataset related to a period of 30 days with a sampling time of 1 min, which is considered sufficient to describe the main dynamics of the process and a wide range of operative conditions. The available processes data refer to various accessible process measures, useful for the plants control and supervision. The measurement of the BF processes state variables is complex and often not feasible, due to the high temperatures involved. For these specific processes, it is difficult to identify significant variables to describe the process itself. For these reasons, starting from a large initial set of variables, the first selection step was manually carried out, through the analysis of the processes themselves and the experience of the operators and plant managers. The following data pre-processing allowed identifying unreliable data through outliers detection techniques [31, 32], and the final inputs set of each model were selected by exploiting a variable selection methodology based on genetic algorithms [33, 34].

The result of the data selection algorithms shows that the main significant variables are the scheduling of the respective process for 2 h ahead, the Boolean information related to the activation of the process itself. The final list of measurement points that characterize the processes and the task objective of this work is presented in Table 1, which provides an overview of the inputs and target variables and related units of measurement (UoM).

**Table 1:** Description of measurement points in the final dataset

Variables	Description	UoM
Scheduling of BFG process	Boolean variable aimed at describing the status of the BF process. On (1), Off (0)	–
Scheduling of Cowpers	Boolean variable aimed at describing the status of the Cowpers. Heating Phase (1), Wind Phase (0)	–
O_2_ content in cold wind	Volume percentage of oxygen in the cold wind input to the BF before heating	%
Cold wind flow	Volume flowrate of cold wind input to the BF before heating	m^3^/h
Hot wind pressure	Pressure of hot wind in input to the BF	bar
BFG production	Volume of produced BF gas	m^3^/h
CO content	Volume percentage of carbon monoxide in BF gas	%
H_2_ content	Volume percentage of hydrogen in BF gas	%
BFG consumption in the Cowpers	Consumption of BFG in Cowpers	m^3^/h
COG consumption in the Cowpers	Consumption of COG in Cowpers	m^3^/h

## Theoretical background

In this section, the main characteristics of the neural network architectures used in this work are introduced, with particular attention to DESNs and LSTMs. LSTMs have been exploited as a valid benchmark for the comparison of the DESNs performances, through a RNN topology with rather effective characteristics in the prediction of short- and long-term nonlinear dynamics, typical of industrial processes.

### Deep Echo-State network architecture

**Fig. 2:**
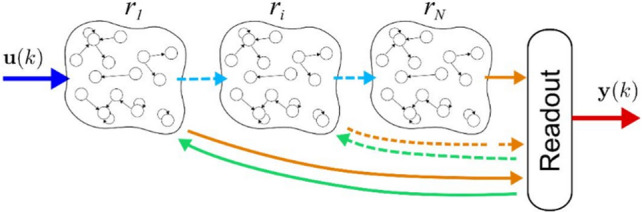
Architecture of a DESN

Among RNNs, ESN topology emerges as a highly efficient tool for reconstructing complex nonlinear dynamics, through the reservoir concept, a particular hidden layer that generates a rich set of dynamics when excited by an exogenous input. This set of dynamics is then composed of a readout layer to generate the input of the network. More in detail, the reservoir acts as a nonlinear filter that enriches the frequency content of the exogenous information to improve regression tasks on a target. As the standard RNNs, this particular architecture is a universal approximator [20] in the case of fading memory input/output system, allowing to reconstruct nonlinear maps with high accuracy. In the last years, the DL paradigm has been exploited also for this particular topology, with the resulted DESN developed by Gallicchio et al. [17, 21] that allows simplifying DL RNN training through algorithms not based on backpropagation routines. The DESN architecture, which is depicted in Fig. 2, is composed of *N* reservoirs ${r}_{i}$ connected in series and a readout that combines the reservoirs dynamics to compute the output of the network.

From a mathematical point of view, the state of DESN ${\varvec{x}}$ is calculated combining the state of each reservoir layer ${{\varvec{x}}}_{i}$ at each time $k$. In particular, the first reservoir ${r}_{1}$ is excited by the exogenous input vector ${\varvec{u}}\left(k\right)$, the state at the previous step $k-1$ and a noise. The following layers are excited by the updated state of the previous reservoir layer ${{\varvec{x}}}_{i-1}\left(k\right)$ and their state at the previous step $k-1$. In general, all the layers of the reservoir can be additionally excited also by a feedback of the network output of the previous instant, as shown in Fig. 2 (in green). For simplicity of discussion, in this work the feedback of the output on the state is not taken into account and is set to zero.

More in detail, the dynamics of the first and following layers are calculated through the update function described by the following equations:

1 $${{\varvec{x}}}_{1}\left(k\right)=f\left({c}_{\mathrm{in}}{{\varvec{W}}}_{{\mathrm{in}}_{1}}{\varvec{u}}\left(k\right) +{{\varvec{W}}}_{{r}_{1}}{{\varvec{x}}}_{1}\left(k-1\right)+{{\varvec{\nu}}}_{1}\left(k\right)\right)$$

2 $${{\varvec{x}}}_{i}\left(k\right)=f\left({c}_{\mathrm{is}}{{\varvec{W}}}_{i{n}_{i}}{{\varvec{x}}}_{i-1}\left(k\right) +{{\varvec{W}}}_{{r}_{i}}{{\varvec{x}}}_{i}\left(k-1\right)+{{\varvec{\nu}}}_{i}\left(k\right)\right)$$

3 $${\varvec{x}}\left(k\right)={\left[\begin{array}{ccc}{{\varvec{x}}}_{1}^{T}\left(k\right)& \cdots & {{\varvec{x}}}_{N}^{T}\left(k\right)\end{array}\right]}^{T}$$

where $i$ is the $i$-th reservoir layer, $f$ is the activation function of the reservoir neurons (typically a tanh function), ${c}_{\mathrm{in}}$ and ${c}_{\mathrm{is}}$ are, respectively, the input scaling and inter-scaling factors, ${{\varvec{W}}}_{{\mathrm{in}}_{1}}$ and ${{\varvec{W}}}_{i{n}_{i}}$ the input matrices of the first and i-th reservoir layer characterized by dimensions ${n}_{1}\times {n}_{\mathrm{in}}$ and ${n}_{i}\times {n}_{\mathrm{i}-1}$, ${n}_{i}$ is the number of neurons of the $i$-th reservoir, ${{\varvec{W}}}_{{r}_{i}}$ is the $i$-th reservoir matrix, ${\varvec{y}}$ is the output of the network, and ${{\varvec{\nu}}}_{i}$ is a small amplitude white noise. The output of the readout is calculated as:

4 $${\varvec{y}}\left(k\right)={f}_{\mathrm{o}}\left({{\varvec{W}}}_{\mathrm{o}}{\varvec{x}}\left(k\right)\right)$$

where ${f}_{o}$ is the activation function of the readout neurons that in time series regression task is typically the identical function, ${{\varvec{W}}}_{\mathrm{o}}$ a ${n}_{\mathrm{o}}\times {n}_{T}$ matrix and ${n}_{T}$ is the total number of reservoir neurons.

As mentioned before, the training algorithm of ESN topology is one of the main effective aspects that characterize this architecture in terms of performance and computational burden, allowing to calculate only the readout weights, unlike the case of standard RNNs. More in detail, the training procedure consists of two sequential phases: the *network initializations* and the *readout training*.

The objective of the first phase is to initialize the reservoir in order to allow it to generate, during the simulation phase, sufficiently rich and stable dynamics; feature that is called contractivity, thanks to which, neurons gradually forget their previous activation. In the case of shallow ESNs (i.e., made up of a single hidden layer of the reservoir), this property has been extensively studied and baptized echo state property (ESP), through works such as that of Yildiz and Jaeger [22]. ESP has been then extended to the case of the DESN in the work of Gallicchio et al. [21]. These works define necessary, sufficient conditions and empirical guidelines for the design of a reservoir characterized of stability. More in detail, in the *initialization phase,* the reservoir matrices ${{{\varvec{W}}}_{r}}_{i}$ are randomly initialized with a sparse ${\widehat{{\varvec{W}}}}_{{r}_{i}}$, with elements defined in the range [−1, 1]. The sparsity of the matrices, defines the percentage number of internal connections of the reservoir neurons, which is typically set below 5%.

In order to design a contractive reservoir, the matrices ${\widehat{{\varvec{W}}}}_{{r}_{i}}$ are normalized with respect to their spectral radius $\rho \left({\widehat{{\varvec{W}}}}_{{r}_{i}}\right)$ and scaled in order to obtain the desired spectral radius ${\stackrel{\sim }{\rho }}_{i}$:

5 $${{{\varvec{W}}}_{r}}_{i}={\tilde{\rho }}_{i}\frac{{\widehat{{\varvec{W}}}}_{{r}_{i}}}{\rho \left({\widehat{{\varvec{W}}}}_{{r}_{i}}\right)}$$

In the case of DESNs, in order to guarantee the ESP, a necessary condition states that the maximum spectral radius between all layers in the reserve must be less than one [21]. This condition can be considered as a guideline to design a contractive reservoir and in general contractivity must be empirically verified. The spectral radius is an important hyperparameter that allows tuning the frequency content of the dynamics generated in the reservoir. In general, there is some parallelism between the stability limit of linear discrete state space systems and the ESP property for ESNs. In particular, in the case of linear state-space systems, the systems are stable if the poles of the transfer function are inside the circumference with a unit radius. A stage of the initialization concerns the parameters and the weights related to the input of each reservoir layer. Even in the case of input matrices, elements are randomly initialized with values in the range [−1, 1]. These matrices are also scaled by additional coefficients ${c}_{\mathrm{in}}$ and ${c}_{\mathrm{is}}$, called input scaling and inter-scaling factors. A last important hyperparameter that must be initialized is related to the amplitude of the noise ${v}_{i}$, whose level can be set with a single value for all the reservoir layers. These factors allow balancing the level of exogenous excitation of each reservoir layer, in such a way as to amplify this contribution or not.

As mentioned before, the *training phase* consists only in the calibration of the readout weights. All the parameters set during the initialization phase are not affected by training routines. In particular, the readout can be trained by minimizing the regression error through non-iterative algorithms that outperform iterative backpropagation routines. A common solution for training the readout is the Tikhonov regularization least square algorithm, which allows calculating weights by minimizing the mean square error on the training dataset:

6 $${{\varvec{W}}}_{\mathrm{o}}=\bar{{\varvec{Y}}}{{\varvec{X}}}^{T}{\left({\varvec{X}}{{\varvec{X}}}^{T}+\lambda {\varvec{I}}\right)}^{-1}$$

where $\stackrel{-}{{\varvec{Y}}}$ and ${\varvec{X}}$ are the sequences of target time series and the state collection matrix the reservoir calculated through Eqs. (1) and (2). $\lambda$ is the regularization coefficient of the Tikhonov algorithm, which allows solving ill-posed matrix inversion.

### Long-Short-term memories

**Fig. 3:**
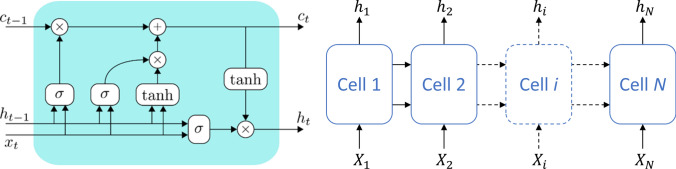
LSTM cell architecture and network example

The long short-term memory (LSTM) introduced by Hochreiter and Schmidhuber [23] has been an important innovation in the RNN field, results of the research on the issues related to the vanishing and exploding gradients that negatively affect the effectiveness of standard RNN architectures. The core of LSTMs are the cells, composed of 4 main subblocks, the *forget gate*, the *input gate*, the *cell state* and the *output gate*. Briefly, the forget gate modulates the information relative to the last step and acts directly on the memory capacity; the input gate modulates the information related to the input that will be stored in the cell; the cell gate is in charge of composing the memory of the LSTM; the output gate calculates the output of the cell. A set of cells (depicted in Fig. 3) can be connected in series to compose a complex network.

## Structure of the models

In this work, the modeling strategy was developed by designing a model for each target variable (the target variables are globally 4), capable of predicting its future evolution for a time window of 2 h ahead, so that each model is particularly specialized in the single task. In a preliminary design stage, several attempts were carried out to develop one single model forecasting all the four variables or two models specialized on two couples of target variables. However, the results in terms of forecasting accuracy were not satisfactory. Moreover, having a higher number of input and output variables, the models were more complex and required a longer time for both training and output calculation. On the other hand, the specialization of each model on a single target variable led to more accurate and simpler model, that also show a higher computational efficiency, which is a relevant aspect for the model implementation within a complex system devoted to optimal POGs management.

The forecasting approach is based on a one-shot multistep manner, so that the *k*-th output of each model predicts the future *k*-th time step. More in detail, the first two models forecast the BFG volume flow and its net calorific value (NCV), the third and the fourth ones, respectively, the consumption of BFG and COG burned in the Cowpers.

**Fig. 4:**
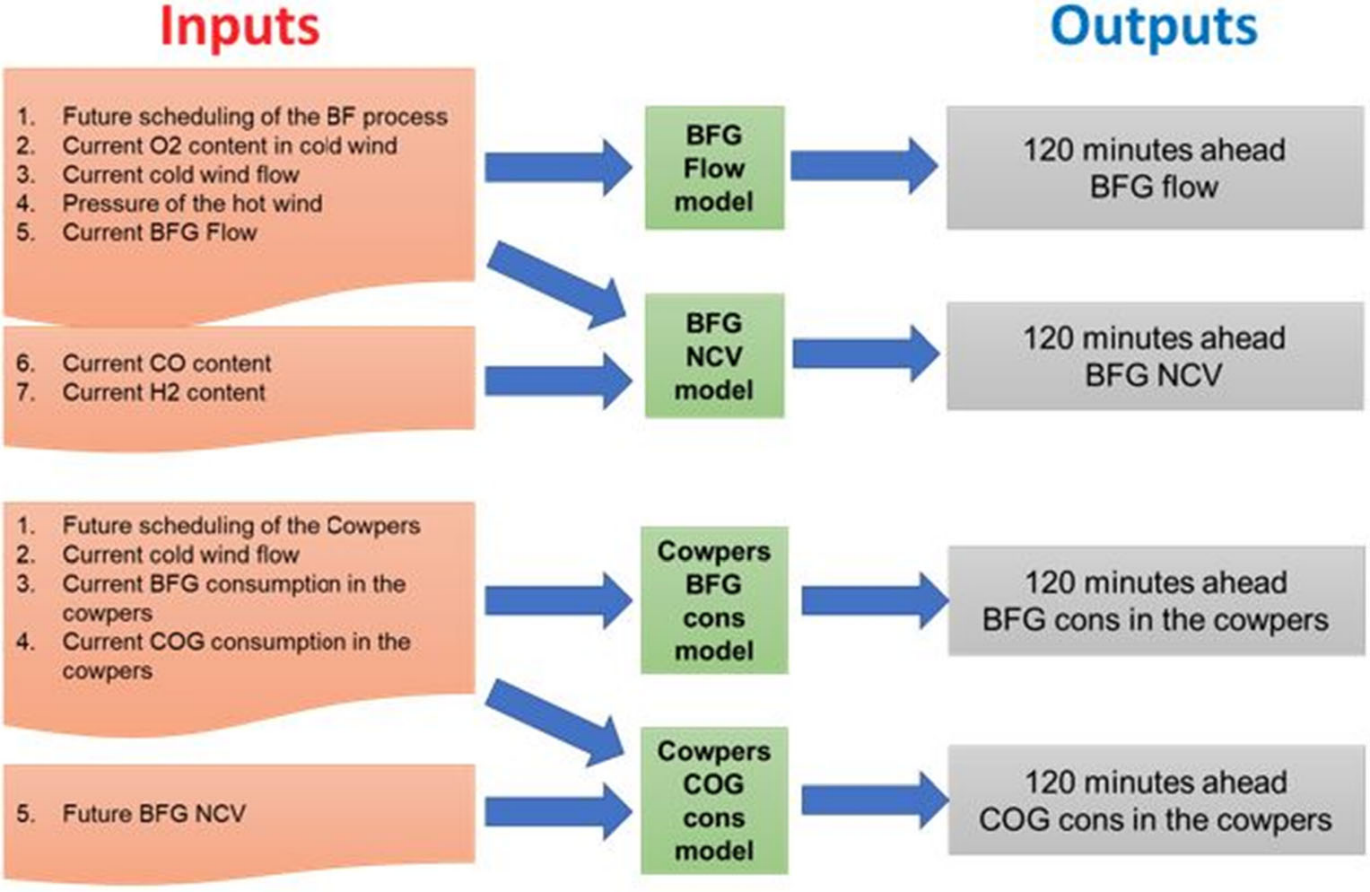
Input/output architecture of the models

The variable routing for each model is described in Fig. 4.

In particular, the first model (BFG Flow) has in input the scheduling of the process, the current O_2_ content measurements in the cold wind, the cold wind volume flow, the pressure of the hot wind and the BFG volume flow. The inputs of the second model (BFG NCV) are the same measurements and in addiction the current BFG CO and H_2_ contents. The inputs of the third model (BFG consumption in the Cowpers) are the Cowpers scheduling and the current measurements of the cold wind flow and the BFG and COG consumption. The inputs of the fourth model (COG consumption in the Cowpers) are the same inputs of the previous model and in addiction the future 2 h predictions of BFG NCV. Therefore, this last model is in cascade to the second one related to BFG NCV.

## Numerical results

The performance of the DESN-based architecture for the considered forecasting tasks has been evaluated in comparison with the ones provided by an LSTM-based architecture, which exploited as a benchmark. In this work, the LSTM architecture is configured as a series of input layer, a LSTM layer, *L*_*LSTM*_ fully connected layers and a linear readout.

The first step of the modeling work has consisted in the definition of the optimal architecture for each task, through the selection of the hyperparameters. In the case of DESN hyperparameters optimization concerns the number of layers *L*_*DESN*_ and neurons of each reservoir ${N}_{R}$ (equal for all the layers), the spectral radius $\rho$ equal for each layer, the input scaling factor ${c}_{in}$ and the inter-layer scaling factor ${c}_{is}$ (equal for each layer). In the case of LSTM, the selection of hyperparameters concerns the number of fully connected layers in series *L*_*LSTM*_ and the number of neurons of each fully connected layer *N*_*LSTM.*_

The selection of the hyperparameters is, in general, a complex topic quite debated by academia; in the case of DESN is in particular an open topic for scientific research. An interesting related work of Gallichio et al. presents some guidelines and recommends some algorithms for their optimization [35]. In this work the hyperparameters optimization exploits a random search technique [36] during which the hyperparameters have been randomly varied with a uniform distribution in the ranges specified later. The search stops 1000 trials with the objective of minimizing the mean value of the *normalized root mean square error (mNRMSE)* of all the outputs of the model, evaluated on the validation set. In this work, *mNRMSE* has been selected as objective function, as it is particularly robust with respect to the *mean absolute percentage error (MAPE)* or other common metrics, thanks to the formulation that considers the overall range amplitude of the targets. Furthermore, the MAPE is not a robust measure where intermittent target values (too many values equal or near to 0) are treated.

7 $${\varvec{m}}{\varvec{N}}{\varvec{R}}{\varvec{M}}{\varvec{S}}{\varvec{E}}=100\frac{1}{{n}_{y}}\sum_{j=1}^{{n}_{y}}\left(\frac{\sqrt{\frac{1}{{N}_{s}}\sum_{k=1}^{{N}_{s}}{\left({\stackrel{-}{y}}_{j}\left(k\right)-{y}_{j}\left(k\right)\right)}^{2}}}{\mathrm{max}\left({y}_{j}\right)-\mathrm{min}\left({y}_{j}\right)}\right)$$

where ${n}_{y}$ is the number of outputs of the network, ${N}_{s}$ the number of samples, ${\stackrel{-}{y}}_{j}$ is the output of the network and ${y}_{j}$ is the target.

For the DESN the hyperparameters are varied in the ranges: number of layers *L*_*DESN*_ = [2 12]; total number of reservoir neurons ${N}_{TOT}=$ [200 2000], with resulting ${N}_{R}=floor\left({N}_{TOT}/{L}_{DESN}\right)$, $\rho =$ [0.1 1]; ${c}_{in}$= [0.01, 10]; ${c}_{is}=$ [0.1 1]; in the case of LSTM: *L*_*LSTM*_ = [1, 10], *N*_*LSTM*_ = [30, 300].

The LSTMs have been trained through the adaptive moment estimation (ADAM) training method [37] in MATLAB environment.

The dataset for each model is composed of the measures of 1 month of operative point, with a sampling time of 1 min, with a total of 43,200 samples. This dataset has been divided in two parts: The first 50% is used for the optimization of hyperparameters and for the following training of the optimal networks, the remaining 50% is used for their test phase. In the hyperparameters selection phase, the first 50% fraction of the overall dataset is divided into 60% training and 40% validation. The dataset fractions used in the training, validation and test phases were selected after a preliminary analysis of the data referring to the associated plant operating conditions. The selected percentages ensure that all the operating phases and process dynamics are meaningfully included in all the data subsets, by making the models robust and accurate when simulating all process phases and dynamics.

The DESN optimal network is summarized in Table 2, which presents the results on the test dataset, while the comparison between the DESN and LSTM optimized models is reported in Table 3, for the training and test dataset.

**Table 2:** Optimal DESN architectures and test results of each model

Model	*L*_*DESN*_	${N}_{R}$	$\rho$	${c}_{in}$	${c}_{is}$	Test *mNRMSE*
BFG Flow BFG NCV Cowpers BFG cons Cowpers COG cons	7 5 5 5	90 139 293 180	0.549 0.991 0.447 0.722	0.154 0.214 5.10 0.073	0.952 0.031 0.022 0.691	6.7 7.3 6.09 9.87

**Table 3:** Comparison between DESN and LSTM architectures

Model	Architecture	Training *mNRMSE*	Test *mNRMSE*
BFG Flow	DESN	5.02	6.70
	LSTM	7.95	10.92
BFG NCV	DESN	5.93	7.31
	LSTM	8.36	16.6
Cowpers BFG consumption	DESN	4.60	6.09
	LSTM	4.95	6.38
Cowpers COG consumption	DESN	8.08	9.87
	LSTM	11.6	13.4

**Fig. 5:**
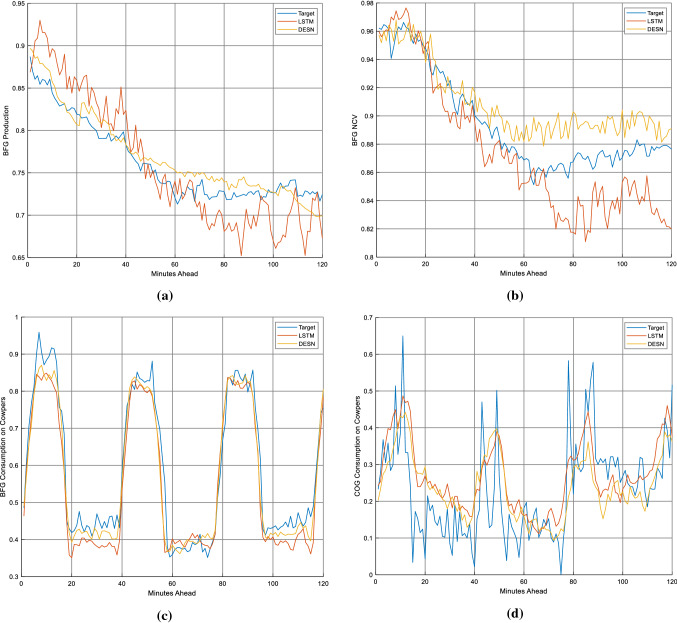
Prediction example of BFG volume flow production (**a**), BFG NCV (**b**), BFG consumption in the Cowpers (**c**) and COG consumption in the Cowpers (**d**)

An example of 2 h ahead prediction of BFG volume flow production and related NCV, COG and BFG consumption in the Cowpers is presented in Fig. 5, where the target is depicted in blue, DESN forecasts in yellow and LSTM ones in orange. These figures show an example of one-shoot multistep prediction of the process behavior for a specific instant of prediction, in which the trends are normalized for confidentiality constraints.

**Fig. 6:**
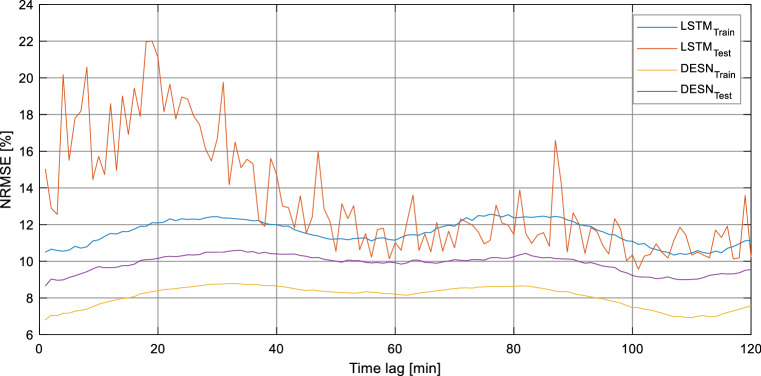
NRMSE trend for LSTM and DESN in function of the forecasting time lag in the case of the model focused on the prediction of COG consumption in Cowpers

Figure 6 shows the behavior of NRMSE as a function of the prediction distance in the case of the fourth model (prediction of COG consumption in Cowpers) and highlights the difference in performance between DESN and LSTM architectures and the oscillatory trend of the error as a function of the prediction distance. The tests show very interesting and encouraging results. In particular, the models that forecasts the BFG production and its NCV are characterized by errors around 7% that, considering the nonlinearity of the problem and the issues related to the measurement of the state of the process, are very low and suitable for control and supervision applications, providing useful information and support to process operators. The results related to the prediction of BFG and COG consumption in the Cowpers are also very satisfactory and also these models can be considered suitable for applications related to control and optimization strategies.

The results show also the difference in performance between DESN and LSTM. In each proposed task, DESNs outperform LSTMs. In more details, DESNs obtains a performance improvement, respectively, of 4.22%, 9.29%, 0.29% and 3.53%, for each task.

**Fig. 7:**
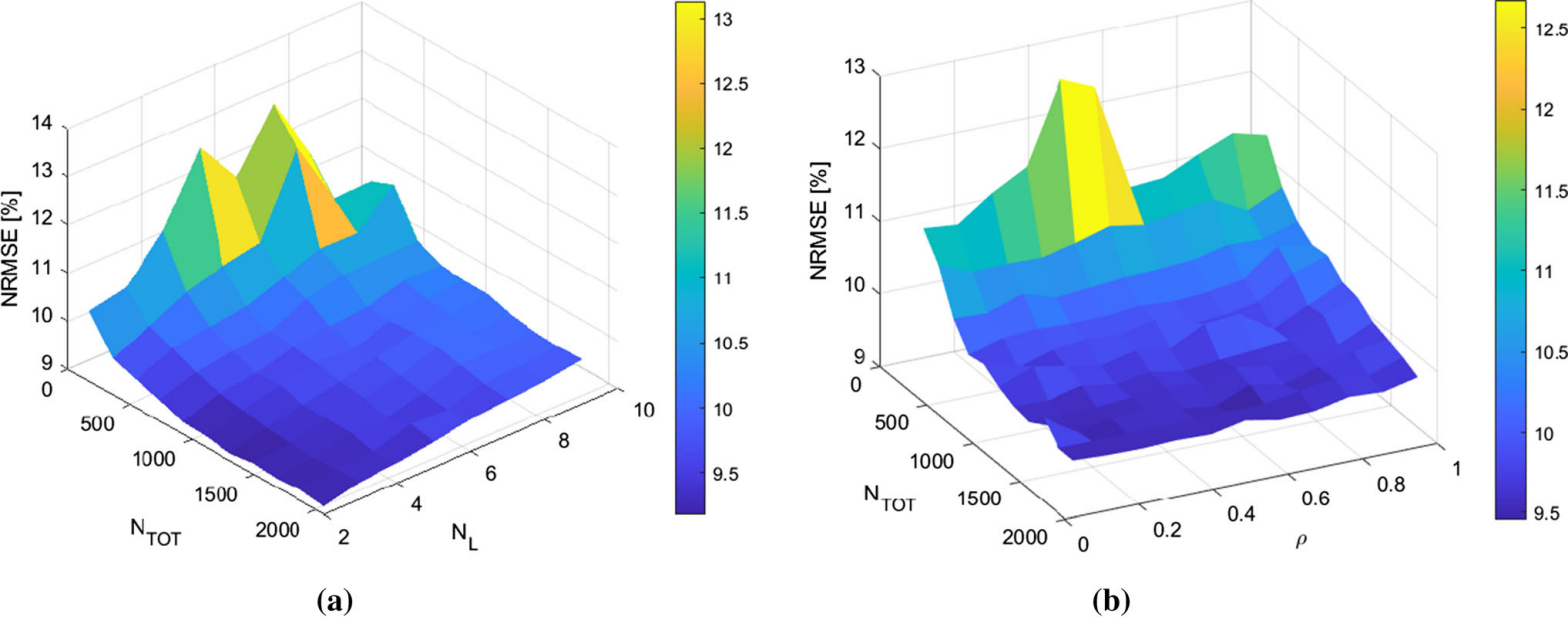
Sensitivity analysis of DESN architecture, in the case of the model focused on the prediction of COG consumption in Cowpers, with respect to: (**a**) number of layers and of total neurons in the network; (**b**) spectral radius and number of neurons

A further study that has been carried out on DESN architectures concerns the sensitivity of the test results to the variation of some of the hyperparameters. The study has been carried out by varying the number of total neurons in the reservoir, the number of layers and the spectral radius, the most important hyperparameters for the training of the network and for which the NRMSE is more sensitive. The other hyperparameters were left constant with respect to the results shown in Table 2. In particular, Fig. 7 shows the results related to the sensitivity analysis on the fourth model (which forecasts the COG consumption in the Cowpers). On the left, the trend of the NRMSE as a function of the number of layers and of the number of total neurons in the network is depicted, while on the right the trend of the NRMSE as a function of the spectral radius and of the number of neurons is shown.

The sensitivity analysis shows several interesting results. Firstly, as expected, the error decrease with increasing numbers of neurons and slightly increases proportionally with the number of layers. The dependence on the radius $\rho$ instead shows a slight convexity. NRMSE is not very sensitive to the spectral radius for a high number of neurons, while it is more sensitive for a low number of neurons. This type of analysis, of course, is to be considered specific for each task and can generally be carried out according to the task to be addressed.

The prediction accuracy is affected not only by the hyperparameters values, but also by some exogenous variables, which are either not available, due to intrinsic lack of adequate monitoring systems, or not transferred in real time and, thus, cannot be exploited in this kind of models. For instance, the process knowledge suggests that an accurate continuous and punctual qualitative and quantitative characterization of the raw materials fed to the BF would be really useful in improving the accuracy of BFG models. However, so far no reliable monitoring systems are available in steelworks, which can provide this kind of information. On the other hand, sometimes the scheduling of plant operation is not fully respected, due to unexpected events (e.g., not scheduled maintenance interventions slowing down the production) or to plant staff decisions. However, such scheduling variations are often not recorded in real time and can be only indirectly inferred from some process variables with a relevant delay, that negatively affect the prediction accuracy. In order to decrease the effect of exogenous variable, improvements are required on the sensing equipment, including development and deployment of monitoring systems which are beyond current state of the art as well as on the ICT systems, which allow fast recording of all scheduling modifications. Furthermore, the implementation of predictive maintenance practices could help avoiding unexpected events, by thus eliminating the root causes of such variations.

**Fig. 8:**
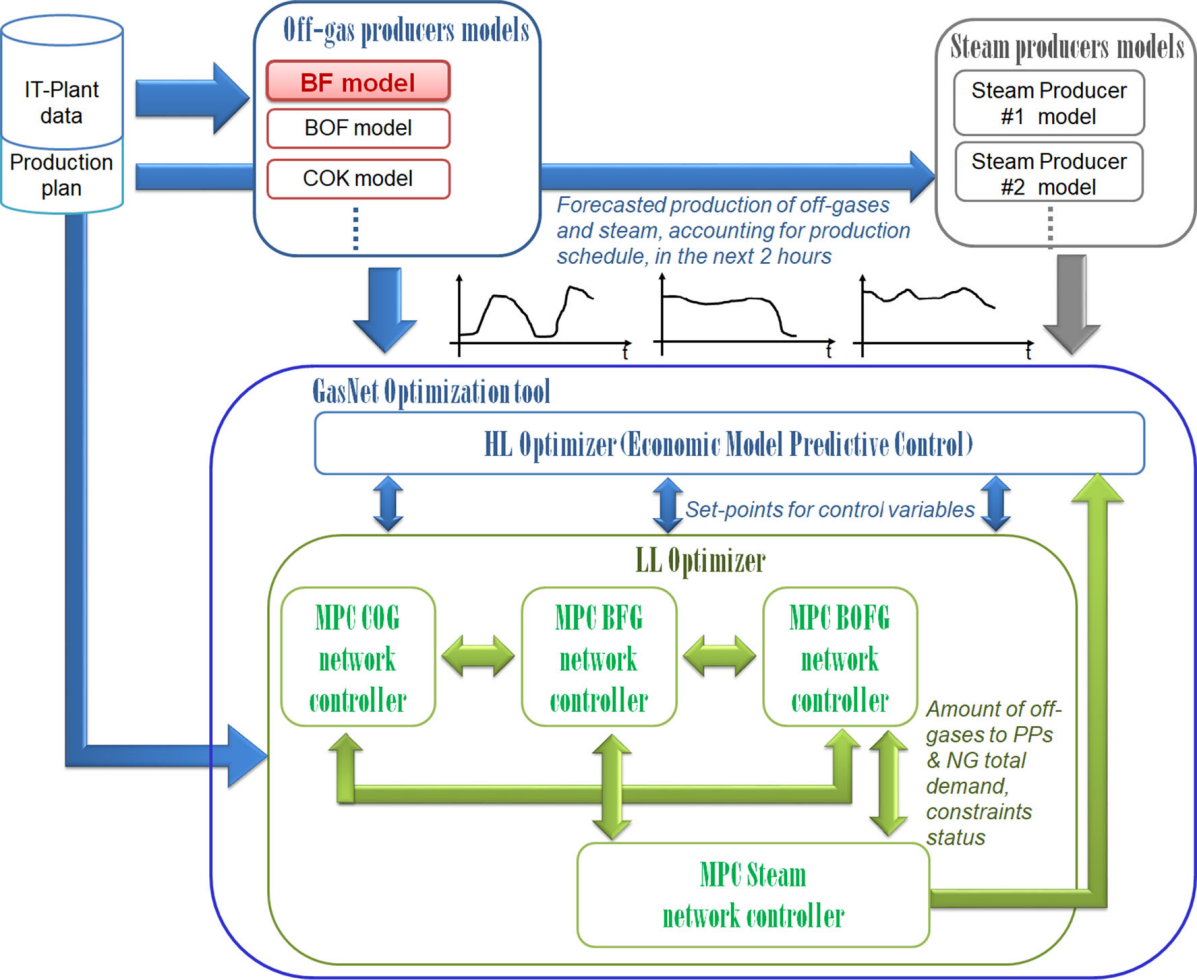
Overall scheme of the gas and steam network management system: the models treated in the present paper lie in the red block

The developed models belong to a model library included in a complex system dedicated to the management of the gas and steam networks in integrated steelworks, where the main POGs and steam producers and consumers are modeled. The 2-h ahead forecasting of POGS consumptions and demands are fed as inputs of an optimizer, which is organized in two levels, such as schematically depicted in Fig. 8.

The high-level optimizer implements a linear programming formulation within a strategy based on the economic model predictive control (MPC), which minimizes the consumptions of natural gas (NG) and purchased electricity and the disposal of excess POG through torches (and emissions, as a consequence), by computing every 15 min the reference set points for the low level. The low-level optimizer distributes the different energy streams over a prediction and control horizon of 2 h, with a control frequency of 1 min, being a distributed controller, which solves in real time a MILP formulation for each specific network through several local economic hybrid MPCs.

The detailed description of this system is out of the scope of the present paper, which is focused on one block of the system that is highlighted in red in Fig. 4. However, being both the high- and the low-level controllers based on MPC, model accuracy is fundamental. Moreover, considering the time constraints and the not negligible number of models and optimization actions to be computed in a single time frame, the computational burden of each single model needs to be affordable, despite the complexity of the processes to model. Finally, the efforts required for the maintenance of the system, including the time required to retune the models with new data, must be limited in order to favor the system practical deployment in the industrial field. The proposed DESN-based model is capable to provide good accuracy at an acceptable computational effort for both computation and tuning and, therefore, represent an ideal solution for the proposed system.

The tests of the system are ongoing, but the preliminary results developed in a German steelworks are very encouraging, showing a potential of drastic reduction of both costs of purchase of NG and electricity (more than 20%) and flares of (more than 60%).

## Conclusions

The paper proposes the application of a particular reservoir computing approach based on DESN in order to model the nonlinear dynamics typical of complex industrial processes. In particular, the problem of forecasting the energetic content of the off-gas produced by the BF, which produces pig iron in integrated steelworks, as well as the consumption of the same gas in the BF Cowpers, is faced. Some DESN-based models have been developed, trained, validated and tested by using real industrial data. The hyperparameters of the DESN-based models are optimized through a random search approach that aims to minimize the validation error. The proposed DESN-based methodology is compared to an LSTM-based architecture in order to assess the accuracy with respect to a well-consolidated state-of-the-art approach. The results show a great advantage in using DESNs to model the dynamic behavior of the considered processes, with respect to the LSTM architecture. The achieved results are satisfactory: The performance of the trained models makes them suitable to an effective integration within a control strategy for the optimal distribution of POGs in the steelworks.

Future work will deal with the integration of the developed models inside a complex decision support system, allowing effective management of gas and steam networks in integrated steelworks.

## Supplementary Information

Below is the link to the electronic supplementary material.

Supplementary file1 (PDF 127 kb)

Supplementary file2 (DOCX 23 kb)
