# Supplementary material for: A Deep Learning-based approach for forecasting off-gas production and consumption in the blast furnace
Source: Neural Comput Appl. 2021 Apr 16;34(2):911–23. doi: 10.1007/s00521-021-05984-x (PMC8051551; doi:10.1007/s00521-021-05984-x)
Supplement: Supplementary file 2 — Supplementary file2 (DOCX 23 kb) [file 521_2021_5984_MOESM2_ESM.docx]

| **List of corrections performed according to the Reviewers’ and Editor’s comments** | | |
| --- | --- | --- |
| **No.** | **Comments** | **Corrections** |
| **Reviewer #1** | | |
| 1 | The theoretical background presented in section 2 is detailed and well written. Both DESNs and LSTMs are presented in detail. To the reviewer's opinion, the only missing thing (in the sense that this could highly improve the value of this section) is to make absolutely clear, instead of the poor starting 4-line note of this section, the problem definition and the available data. This reviewer recommends the following structure (which needs only restructuring of the existing text):   1. Problem definition and adequate description of the needs of steelmaking sites in this respect. This is current section 3 plus a paragraph making inputs and outputs of this problem crystal clear. This could be the first sentence in Section 4 plus a mathematical description of I/Os. For instance, table 4 should move ahead. 2. Theory of DESNs and LSTMs, this already exists and is current section 2. 3. Applications & comparisons…. | The paper structure was amended: the former Section 3 became Section 2 and a description of the available inputs and outputs was included, by moving Table 1 at the end of this section, according to the suggestion of the reviewer. As a consequence, the former Section 2 became Section 3. |
| 2 | Introduction: Although known, define ICT. | It was defined. |
| 3 | Introduction: Suggestion to eliminate the statement "…a certain level of reluctance is shown by plant operators towards novel and more complex control and supervision methodologies", though I personally have the same experience. | The statement was eliminated. |
| 4 | Introduction: Change "gradient descend" to "gradient descent". | It was corrected |
| 5 | Section 4: Try to make the reason we need one model per output clear. | In a very first design stage, the attempt to develop one DESN-based model forecasting all the variables or two models specialized on two couples of variables was made, but it led to worse results in terms of accuracy and required higher computational efforts for both training and computation. In order to clarify this aspect, the following sentence was added in the first paragraph of Section 4:  *“In a preliminary design stage, several attempts were carried out to develop one single model forecasting all the four variables or two models specialized on two couples of target variables. However, the results in terms of forecasting accuracy were not very good. Moreover, having a higher number of input and output variables, the models were more complex and required a longer time for both training and output calculation. On the other hand, the specialization of each model on a single target variable led to more accurate and simpler model, that also show a higher computational efficiency, which is a relevant aspect for the model implementation within a complex system devoted to optimal POGs management.”* |
| 6 | Section 5: Percentages of dataset associated to training and testing sound arbitrary enough. Please explain. | In order to clarify this aspect, the following sentence was included in Section 5 before Table 2:  *“The dataset fractions used in the training, validation and test phases were selected after a preliminary analysis of the data referring to the associated plant operating conditions. The selected percentages ensure that all the operating phases and process dynamics are meaningfully included in all the data subsets, by making the models robust and accurate when simulating all process phases and dynamics.*” |
| 7 | Section 5: DESNs outperform LSTMs; we see this in figs. 5, 6, etc. However, next to this comparison with targets, it would be nice to have a discussion on which other problem parameters (not taken into account in this modelling - I am not referring to hyperparameters etc, I am referring to exogenous parameters) may affect predictions. | Actually some exogenous reasons can affect the prediction, such as correctly highlighted by the Reviewer. In order to clarify this aspect and to answer also to the first comment provided by Reviewer 2, the following paragraph was included in Section 5 after the hyperpameters sensitivity analysis:    *“The prediction accuracy is affected not only by the hyperparameters values, but also by some exogenous variables, which are either not available, due to intrinsic lack of adequate monitoring systems, or not transferred in real time, and, thus, cannot be exploited in this kind of models. For instance, the process knowledge suggests that an accurate continuous and punctual qualitative and quantitative characterization of the raw materials fed to the BF would be really useful in improving the accuracy of BFG models. However, so far no reliable monitoring systems are available in steelworks, which can provide this kind of information. On the other hand, sometimes the scheduling of plant operation is not fully respected, due to unexpected events (e.g. not scheduled maintenance interventions slowing down the production) or to plant staff decisions. However, such scheduling variations are often not recorded in real time and can be only indirectly inferred from some process variables with a relevant delay, that negatively affect the prediction accuracy. In order to decrease the effect of exogenous variable, improvement are required on the sensing equipment, including development and deployment of monitoring systems which are beyond current state of the art as well as on the ICT systems, which allow fast recording of all scheduling modifications. Furthermore, the implementation of predictive maintenance practices could help avoiding unexpected events, by thus eliminating the root causes of such variations.”* |
| **Reviewer #2** | | |
| 1 | In section 4, the procedure followed to create the dataset is described, mentioning the difficulty of carrying out the required measurements and selecting the significant variables. In section 5, is mentioned that the models (with errors varying from 6.09% to 9.87%) can be used for control. Since the database is very important for the prediction capability of a network, what part of the errors can be attributed to these uncertainties? Are there other alternatives? | In order to clarify this aspect and to answer also to last comment by Reviewer 1, the following paragraph was included in Section 5 after the hyperpameters sensitivity analysis:    *“The prediction accuracy is affected not only by the hyperparameters values, but also by some exogenous variables, which are either not available, due to intrinsic lack of adequate monitoring systems, or not transferred in real time, and, thus, cannot be exploited in this kind of models. For instance, the process knowledge suggests that an accurate continuous and punctual qualitative and quantitative characterization of the raw materials fed to the BF would be really useful in improving the accuracy of BFG models. However, so far no reliable monitoring systems are available in steelworks, which can provide this kind of information. On the other hand, sometimes the scheduling of plant operation is not fully respected, due to unexpected events (e.g. not scheduled maintenance interventions slowing down the production) or to plant staff decisions. However, such scheduling variations are often not recorded in real time and can be only indirectly inferred from some process variables with a relevant delay, that negatively affect the prediction accuracy. In order to decrease the effect of exogenous variable, improvement are required on the sensing equipment, including development and deployment of monitoring systems which are beyond current state of the art as well as on the ICT systems, which allow fast recording of all scheduling modifications. Furthermore, the implementation of predictive maintenance practices could help avoiding unexpected events, by thus eliminating the root causes of such variations”* |
| 2 | In section 4, the authors mention that for each target one model is designed. From fig. 4 it can be shown that the inputs between the BFG Flow and NCV models and those between the Cowpers BFG and COG cons models differ only at the 6th, 7th and at the 5th component, respectively. Could the number of models be smaller for simplicity and application reasons (meaning to create one model for the 1st and 2nd target and a second one for the 3rd and 4th) maintaining the same accuracy? Is the DESN architecture capable of that? | In a very first design stage, the attempt to develop one DESN-based model forecasting all the variables or two models specialized on two couples of variables was made, but it led to worse results in terms of accuracy and required higher computational efforts for both training and computation. In order to clarify this aspect, the following sentence was added in the first paragraph of Section 4:  *“In a preliminary design stage, several attempts were developed to develop one single model forecasting all the four variables or two models specialized on two couples of target variables. However, the results in terms of forecasting accuracy were not very good. Moreover, having a higher number of input and output variables, the models were more complex and required a longer time for both training and output calculation. On the other hand, the specialization of each model on a single target variable led to more accurate and simpler model, that also show a higher computational efficiency, which is a relevant aspect for the model implementation within a complex system devoted to optimal POGs management.”* |
| 3 | In section 3, the authors describe the industrial application they are dealing with, mentioning the importance of predicting POGs production and consumption in reducing cost and emissions. In section 5, the authors present the application of the DESN architecture in the aforementioned forecasting. However, the benefit of using this architecture in terms of energy cost and emission is not clear. Could the authors calculate or estimate this reduction and the gain of using the DESN architecture? | Some paragraphs have been added at the end of Section 5, including one Figure, in order to describe the context in which the described models are exploited and the importance of adopting the proposed DESN-based architecture.  Some figures on savings obtained in some preliminary tests are also provided. |
| 4 | In p. 3, line 29 ICT is not defined. | It was defined |
| 5 | In p. 12, lines 25-26 "concerns the of number of the layers". | It was corrected |
| 6 | In p. 13, a typo in NL=floor (NTOT/NL). | It was corrected |
| 7 | A prediction example of BFG Flow and NVC could be possibly added in figs 5 and 6. | Two figures have been added in Section 5 in order to provide an example of BFG volume flow production and NCV predictions.  The four figures concerning the provided forecasting have been gouped in one single figure, i.e. Figure 5. |
| 8 | In p. 15, lines 22 and 59 Figure 7 appears twice, instead of Figure 7 and Figure 8. | It was corrected |
| 9 | The titles of figs 2,5,6,7,(8?) and tables 1,2 end with ".", while those of figs 1,3,4 and table 3 do not. | The style of the captions of all figures and tables was made homogeneous. |
| 10 | In citation [12], the Journal name is the only one in abbreviation format. | It was corrected |
| **Other changes** | | |
| 1 | Some minor amendments of the English language have been performed | |
| 2 | The captions of Figures 7 and 8 have been modified in order to improve and clarify the description of the figures. | |

The authors wish to thank the Reviewers for the fruitful comments that allowed improving the paper and clarifying some major aspects of the developed work.
