# Supplementary material for: A Deep Learning-based approach for forecasting off-gas production and consumption in the blast furnace
Source: Neural Comput Appl. 2021 Apr 16;34(2):911–23. doi: 10.1007/s00521-021-05984-x (PMC8051551; doi:10.1007/s00521-021-05984-x)
Supplement: Supplementary file 1 — Supplementary file1 (PDF 127 kb) [file 521_2021_5984_MOESM1_ESM.pdf]

INSTITUTE OF COMMUNICATION, INFORMATION AND PERCEPTION TECHNOLOGIES

Sant'Anna
School of Advanced Studies – Pisa

Pisa, December 4th 2020

Submission of an article to be considered for possible publication on Neural Computing and Applications

Dear Editor(s),

Following the kind invitation which was sent by Prof. Lazaros Iliadis on September 14, to be considered for potential publication in the Neural Computing and Applications Special Issue *"Topical Collection on Advances of Neural Computing phasing challenges in the era of 4th industrial revolution"*.
The paper is entitled "*A Deep Learning-based approach for forecasting off-gas production and consumption in the blast furnace*", is authored by myself and my colleagues Ismael Matino, Valentina Colla and Ramon Speets.
This paper is an extended version of the paper entitled "Deep Echo State Networks in Industrial Applications", which was presented by myself within the 16th International Conference on Artificial Intelligence Applications and Innovations AIAI 2020, which was held in Greece in June 2020.
The paper shows how Deep Learning can be effectively applied to the problem of forecasting the production and internal consumption of Blast Furnace off-gas on a two hours-wide horizon.

Yours Faithfully
Dr. Ing. Stefano Dettori– Corresponding Author

Assistant Researcher
Scuola Superiore Sant'Anna- TeCIP Institute
Center ICT for Complex Industrial Systems and Processes (ICT-COISP)
Via Moruzzi 1, 56124 Pisa, ITALY
Tel: +39 050 882330
e-mail: s.dettori@santannapisa.it
